# Implications of the KHDC4-TRAF2 axis in the context of prostate cancer prognosis

**DOI:** 10.18632/aging.206273

**Published:** 2025-06-23

**Authors:** Su-Wei Hu, Chia-Chang Wu, Shao-Wei Dong, Kai-Yi Tzou, Chih-Heng Chen, Yuan-Hung Wang, Yen-Nien Liu, Chiao-Chun Liao, Chien-Hsiu Li

**Affiliations:** 1 Department of Urology, Shuang Ho Hospital, Taipei Medical University, New Taipei City, Taiwan; 2 Taipei Medical University (TMU) Research Center of Urology and Kidney, Taipei Medical University, Taipei City, Taiwan; 3 Department of Urology, School of Medicine, College of Medicine, Taipei Medical University, Taipei City, Taiwan; 4 Graduate Institute of Clinical Medicine, College of Medicine, Taipei Medical University, Taipei City, Taiwan; 5 Department of Medical Research, Shuang Ho Hospital, Taipei Medical University, New Taipei City, Taiwan; 6 Graduate Institute of Cancer Biology and Drug Discovery, College of Medical Science and Technology, Taipei Medical University, Taipei City, Taiwan; 7 Department of Tropical Medicine, School of Medicine, College of Medicine, National Yang Ming Chiao Tung University, Taipei City, Taiwan

**Keywords:** prostate cancer, KHDC4, TRAF2, bioinformatic, prognosis

## Abstract

The inability to effectively identify the formation of advanced-stage tumors poses a challenge in precisely determining when to intervene in prostate cancer (PCa). Despite the use of PSA as a screening factor, it still falls short in significantly improving the diagnosis and prognosis of advanced PCa. Identifying novel prognosis biomarkers to assist in confirming the progression of advanced PCa will contribute to more precise and effective therapeutic approaches. Through a comparative analysis between late-stage and early-stage TCGA-PRAD transcriptomes, KHDC4 has been identified as a key and specific member of the KHDC family that shows increased expression in PCa. The elevated levels of KHDC4 in late-stage and lymph node metastasis are positively correlated with poorer overall survival and disease-free survival rates in PCa patients. Simulated molecular regulation networks and *in vitro* results support the notion that the KHDC4-TRAF2 axis contributes to tumor malignancy features in late-stage and lymph node metastasis tumor samples, consequently correlating with worse progression-free interval and disease-free interval prognosis values in TCGA-PRAD. It is noteworthy that the positive correlation of the distribution of KHDC4 and TRAF2 with the Gleason score is superior to that of KLK3. Promoter analysis reveals that KHDC4 and TRAF2 share a similar upstream regulator, E2F4, for their transactivation. Molecular simulated profiles, mimicking downstream effectors under both KHDC4 and TRAF2 regulation, can be utilized as signatures for overall survival and disease-free survival prognosis purposes. In conclusion, this systematic analysis study indicates that the axis of KHDC4-TRAF2 may serve as a valuable prognostic model for evaluating advanced PCa.

## INTRODUCTION

According to Rebecca L. Siegel’s long-term analysis of annual cancer incidence in the United States, it is revealed that PCa has consistently ranked first in the past five years. From 2020 to 2024, the incidence in the male population increased from 21% to 29%, while the mortality rate rose from 10% to 11%, placing it second only to lung cancer [1, 2]. Although the detection method of prostate-specific antigen (PSA) improves PCa diagnosis, its accuracy is still influenced by non-tumor-related symptoms in the prostate. Therefore, there is a necessity to develop novel adjunctive prognosis biomarkers. Given that hormone therapy was discovered in 1942 as an effective means to block PCa progression, it has become the primary treatment strategy for patients, with over 70% showing significant responses in the early stages [3, 4]. However, despite this, a majority of patients still develop castration-resistant prostate cancer (CRPC) after undergoing androgen deprivation. Although abiraterone and enzalutamide are employed as antagonists targeting androgen biosynthesis and its receptor, leading to further extension of CRPC patients’ survival, they are accompanied by the formation of drug resistance. Therefore, the development of alternative treatments as a strategy for addressing advanced PCa becomes imperative for overcoming this challenge.

Heteronuclear ribonucleoprotein K homology domain-containing protein 4 (KHDC4) is also known as KIAA0907 or BLOM7. According to the Alliance of Genome Resources databases, the initial analysis of gene ontology annotates KHDC4 with RNA binding activity [5]. Yeast two-hybrid experiments have demonstrated that the alpha form of KHDC4 promotes the localization of the CDC5L-SNEV (Prp19-Pso4) complex to mRNA splice sites, thereby enhancing various pre-mRNA splicing activities [6]. In HeLa cells, the AC-rich RNA aptamer can interact with KHDC4 alpha to form pre-mRNA splicing catalysis [7]. An increased level of KHDC4 has been observed in colorectal cancer tissues and is correlated with a worse survival rate. Knockdown of KHDC4 suppresses the proliferation and migration activity of colorectal tumor cells [8]. Single-cell RNA sequencing-based computational analysis suggests that the distribution of KHDC4 associated with the binding affinity of oxfendazole and mevastatin in advanced osteosarcoma can serve as a potential model for predicting treatment outcomes [9]. Interestingly, in lung cancer or PCa, the transcript of KHDC4 is found to undergo alternative splicing at the 10th intron, producing small nucleolar RNA 42 (SNORA42). siRNA-based loss of function on SNORA42 has been shown to inhibit tumor growth in lung or PCa [10, 11]. In breast cancer, the expression of KHDC4 is involved in the regulation by miR-641, contributing to tumor malignancy [12]. However, the prognostic role and molecular mechanism of KHDC4 in PCa remain unclear.

The Tumor Necrosis Factor Receptor Associated Factor 2 (TRAF2) is classified within the TNF receptor-associated factor (TRAF) protein family. TRAF2 functions as a stress response protein, participating in programmed cell death, autophagy, and ER stress processes. The protein-protein interaction between TRAF2 and TRADD can activate NF-κB signaling to counteract apoptotic and non-canonical NF-κB events [13–18]. The residual Thr117 of TRAF2 in the RING-type zinc finger domain has been identified as necessary for TNF-α induced JNK or NF-κB signaling transduction and has been developed as an inhibitor strategy [19]. The expression of TRAF2 is implicated in Epstein-Barr virus infection by interacting with LMP1 and Na protein, contributing to nasopharyngeal carcinoma or gastric cancer oncogenesis [20]. In multiple myeloma and lymphoma, the negative regulation of TRAF2 has been found to assist in inhibiting oncogenesis by suppressing non-canonical NF-κB signaling activation [20]. The interaction of TRAF2 with different protein partners has been identified to participate in the development of various cancer cells. In colorectal cancer, β-catenin protein stability can be stabilized and activate Wnt signaling through its interaction with TRAF2 [20]. However, the relationship between TRAF2 and KHDC4 remains unclear.

In this study, an analysis of the transcriptome profiles of TCGA-PRAD identified the increased expression of KHDC4 and TRAF2 in the late stage, serving as biomarkers for distinct prognostic approaches. The positively correlated upstream and downstream effectors of the KHDC4-TRAF2 axis could be employed as a signature for the prognosis of advanced PCa.

## RESULTS

### Elevated KHDC4 levels are associated with the progression of late-stage prostate cancer

**Figure 1:**
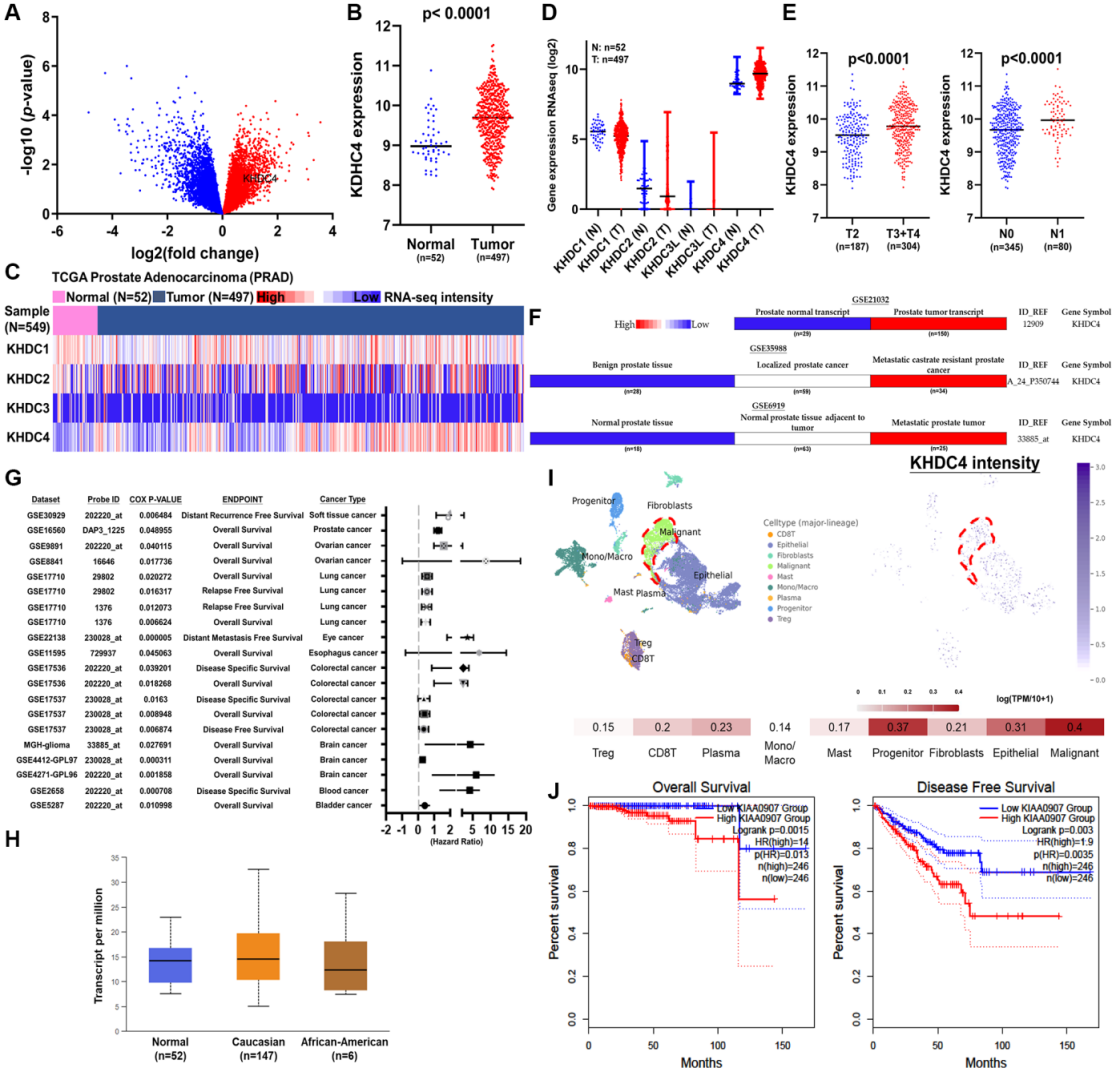
**Increased KHDC4 levels are associated with the development of advanced prostate cancer.** (**A**) The volcano plot illustrates an elevation in KHDC4 levels among patients in the late stage of TCGA-PRAD. (**B**) The associated expression intensity of KHDC4 in patients from TCGA-PRAD. (**C**) The correlated expression levels of KHDC members in patients from TCGA-PRAD. (**D**) The correlated expression intensity of KHDC4 in normal solid tissue and primary prostate tumor. (**E**) The difference in the level of KHDC4 between late-stage or lymph node metastasis in TCGA-PRAD patients. (**F**) The correlation of KHDC4 levels with advanced prostate cancer across different sources of prostate cohorts (GSE21032, GSE35988, GSE6919). (**G**) The correlation of KHDC4 levels with prognosis values in cancer across different cohort sources. (**H**) KHDC 4 expression levels across diverse racial backgrounds. (**I**) Analysis of single-cell sequencing profiles (GSE176031) reveals the relative expression intensity of KHDC4 across different cell types. (**J**) The prognostic impact of KHDC4 on overall survival and disease-free survival rates in prostate cancer.

To identify novel biomarkers capable of distinguishing early-stage from late-stage PCa, transcriptome files related to the pathological T classification according to the 7th edition of AJCC, ranging from tumor confined within the prostate (pathological T2a stage) to tumor invading adjacent structures of pathological pT4 stage, were obtained from The Cancer Genome Atlas Program (TCGA) – prostate adenocarcinoma (PRAD). A volcano plot was utilized to profile genes that exhibited differential and increased expression in pathologic T4 stage compared to T2a (Figure 1A) (Supplementary Table 1). Among approximately 7,828 upregulated genes in the T4 stage, KHDC4 (KIAA0907) was identified as a potential novel biomarker for PCa progression. In TCGA-PRAD patients, KHDC4 exhibited a significant increase in tumor groups compared to normal groups (*p* < 0.0001) (Figure 1B) (Supplementary Table 2). Currently, four KH domain-containing protein members have been identified. A heatmap profile illustrates their respective related expression intensities in TCGA-PRAD patients (Figure 1C) (Supplementary Table 3). Based on their related expression, the distribution of KHDC4 was the most highly expressed member and increased in the tumor group (*p* < 0.0001) (Figure 1D) (Supplementary Table 3). Furthermore, higher KHDC4 expression was observed in advanced invasive stages (pathologic T3+T4) and regional lymph node metastasis stage (pathologic N1) (*p* < 0.0001) (Figure 1E) (Supplementary Table 4). Consistently, a similar trend was obtained through the analysis of PCa-related cohorts, including Taylor (GSE21032), Tomlins (GSE35988), and Monzon (GSE6919) cohorts (Figure 1F) (Supplementary Table 5). From a pan-cancer perspective, KHDC4 was found to be increased in multiple cancer types (Supplementary Figure 1) and correlated with worse prognosis values (Figure 1G) (Supplementary Table 6), including PCa. Importantly, the expression of KHDC4 did not significantly vary across different ethnicities (*p* = ns) (Figure 1H). Notably, a single-cell level dataset (GSE176031) profiling the distribution of KHDC4 revealed that malignant cell types (highlighted in red boxes) exhibited the highest intensity level (0.4 (log (TPM/10+1))) compared to other major cell lineages in the tumor microenvironment (Figure 1I). Using KHDC4 as a prognostic factor for PCa patients demonstrated that those with higher KHDC4 expression had a worse overall survival rate (*p* = 0.0015, HR = 14) and disease-free survival rate (*p* = 0.003, HR = 1.9) (Figure 1J). To investigate the role of KHDC4 in driving malignancy in PCa, we generated KHDC4 knockdown models using the C4-2 and PC-3 cell lines. The effectiveness of KHDC4 silencing was confirmed by qPCR (Supplementary Figure 2A), with subsequent immunoblotting assays verifying a significant decrease in KHDC4 protein levels (Supplementary Figure 2B). Functional assays revealed that KHDC4 knockdown led to a notable reduction in tumor growth rates, as evidenced by colony formation and cell proliferation assays (Supplementary Figure 2C, 2D). Moreover, KHDC4 depletion significantly impaired tumor cell motility, as demonstrated by wound healing assays (Supplementary Figure 2E). The migratory and invasive capacities of the PCa cells, assessed through fibronectin and Matrigel-coated Boyden chambers, were also markedly diminished following KHDC4 knockdown (Supplementary Figure 2F, 2G). These results underscore the critical role of KHDC4 in PCa tumor progression and highlight its potential as a prognostic biomarker for advanced disease.

### TRAF2 is implicated in KHDC4-mediated adverse prognosis outcomes in prostate cancer

**Figure 2:**
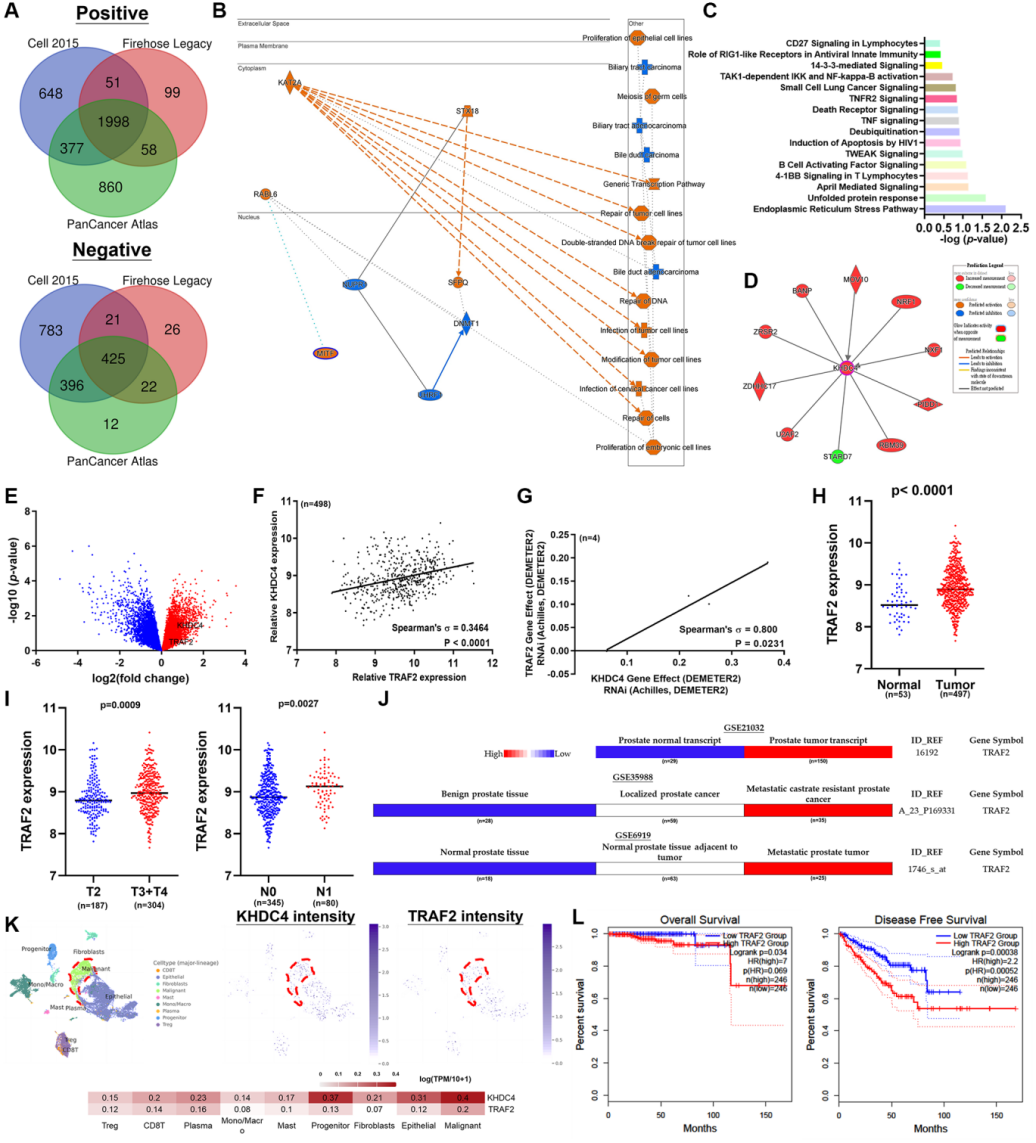
**KHDC4-mediated adverse prognosis outcomes in prostate cancer are linked to the involvement of TRAF2.** (**A**) A Venn diagram analysis was conducted to gather molecules that are positively or negatively correlated with KHDC4 in TCGA-PRAD datasets, including Cell 2015, Firehose Legacy, and PanCancer Atlas. (**B**) The graphical abstract illustrates the potential biological roles influenced by KHDC4 in prostate cancer. (**C**) The canonical pathways influenced by KHDC4-related molecules. (**D**) Molecular connections associated with KHDC4 regulation in prostate cancer. (**E**) Volcano plot depicts elevated TRAF2 levels in late-stage TCGA-PRAD patients. (**F**) The correlation between KHDC4 and TRAF2 in TCGA-PRAD patients. (**G**) The correlation between KHDC4 and TRAF2 in CCLE prostate cancer cell lines. (**H**) The related expression intensity of TRAF2 in TCGA-PRAD patients. (**I**) The difference in the level of TRAF2 between late-stage or lymph node metastasis in TCGA-PRAD patients. (**J**) The correlation of TRAF2 levels with advanced prostate cancer in different sources of prostate cohorts (GSE21032, GSE35988, GSE6919). (**K**) Analysis of single-cell sequencing profile (GSE176031) to examine the relative expression levels of KHDC4 and TRAF2 across different cell types. (**L**) The impact of TRAF2 expression levels on overall survival and disease-free survival rates in prostate cancer.

To further elucidate the primary molecular mechanisms underlying KHDC4-mediated malignancy in PCa, a simulated molecular regulation network based on KHDC4 correlations was established using three distinct TCGA-PRAD transcriptome profiles from Cell 2015, Firehose Legacy, and PanCancer Atlas datasets [21–24]. A total of 1998 positively (Spearman values > +0.3) or 425 negatively (Spearman values > −0.3) correlated molecules with KHDC4 were selected by Venn diagrams across the three TCGA-PRAD datasets (Figure 2A) (Supplementary Table 7). Graphical summaries generated from IPA (Ingenuity Pathway Analysis) software revealed that KHDC4 primarily influences cellular functions, with a major involvement in proliferation and repair processes (Figure 2B). These biological functions are likely mediated by various signaling transductions regulated by KHDC4, as listed in Figure 2C (Supplementary Table 8). A molecular interaction analysis supports the notion that this simulated molecular model can identify numerous genes reported to be involved in KHDC4 regulation (Figure 2D). Notably, according to the gene ontology results, TRAF2 was identified as participating in each potential signaling pathway (Figure 2C) (Supplementary Table 8). Similar to KHDC4, TRAF2 also exhibited an increase in expression in pathologic T4 stage compared to T2a in the volcano plot (Figure 2E) (Supplementary Table 1). The related expression levels of KHDC4 and TRAF2 were significantly positive in TCGA-PRAD (Spearman’s correlation = 0.3464, *p* < 0.0001) (Figure 2F) (Supplementary Table 4). This relationship was also reflected in PCa cell lines according to the CCLE (Cancer Cell Line Encyclopedia) database (Spearman’s correlation = 0.800, *p* = 0.0231) (Figure 2G) (Supplementary Table 9). The expression of TRAF2 was increased in the tumor group compared to normal (*p* < 0.0001) (Figure 2H) (Supplementary Table 2). PCa patients in advanced invasive stages (pathologic T3+T4) (*p* < 0.0009) and regional lymph node metastasis stage (pathologic N1) (*p* < 0.0027) exhibited higher TRAF2 expression (Figure 2I) (Supplementary Table 4). Consistently, a similar trend was observed in PCa-related cohorts, including Taylor (GSE21032), Tomlins (GSE35988), and Monzon (GSE6919) cohorts (Figure 2J) (Supplementary Table 5). From a pan-cancer perspective, TRAF2 was increased in multiple cancer types (Supplementary Figure 3), including PCa. Similar to KHDC4, the distribution of TRAF2 indicated a malignant cell type (highlighted in red) with the highest intensity level compared to other major cell lineages in the PCa tumor microenvironment (GSE176031) (Figure 2K). Using TRAF2 as a prognostic factor for PCa patients revealed that higher TRAF2 levels were associated with worse overall survival rate (*p* = 0.034, HR = 7) and disease-free survival rate (*p* = 0.00038, HR = 2.2) (Figure 2L).

To assess the role of TRAF2 in PCa, knockdown models were developed using C4-2 and PC3 cell lines. The successful knockdown of TRAF2 was validated through qPCR (Supplementary Figure 4A) and immunoblotting (Supplementary Figure 4B). Our findings showed that reducing TRAF2 levels led to a significant decrease in colony formation (Supplementary Figure 4C) and cellular proliferation (Supplementary Figure 4D), aligning with previous research that implicates TRAF2 in tumor progression [25]. Furthermore, TRAF2 depletion impaired the migratory (Supplementary Figure 4E, 4F) and invasive (Supplementary Figure 4G) capabilities of PCa cells, emphasizing its critical role in tumor malignancy. These results demonstrate that the correlation-based simulated molecular regulation can reflect the potential role of KHDC4 in PCa, and the positive correlation of pathologic features linking TRAF2 involvement in KHDC4-mediated PCa malignancy.

### The pathological correlation between KHDC4 and TRAF2 contributes to an advanced Gleason score

**Figure 3:**
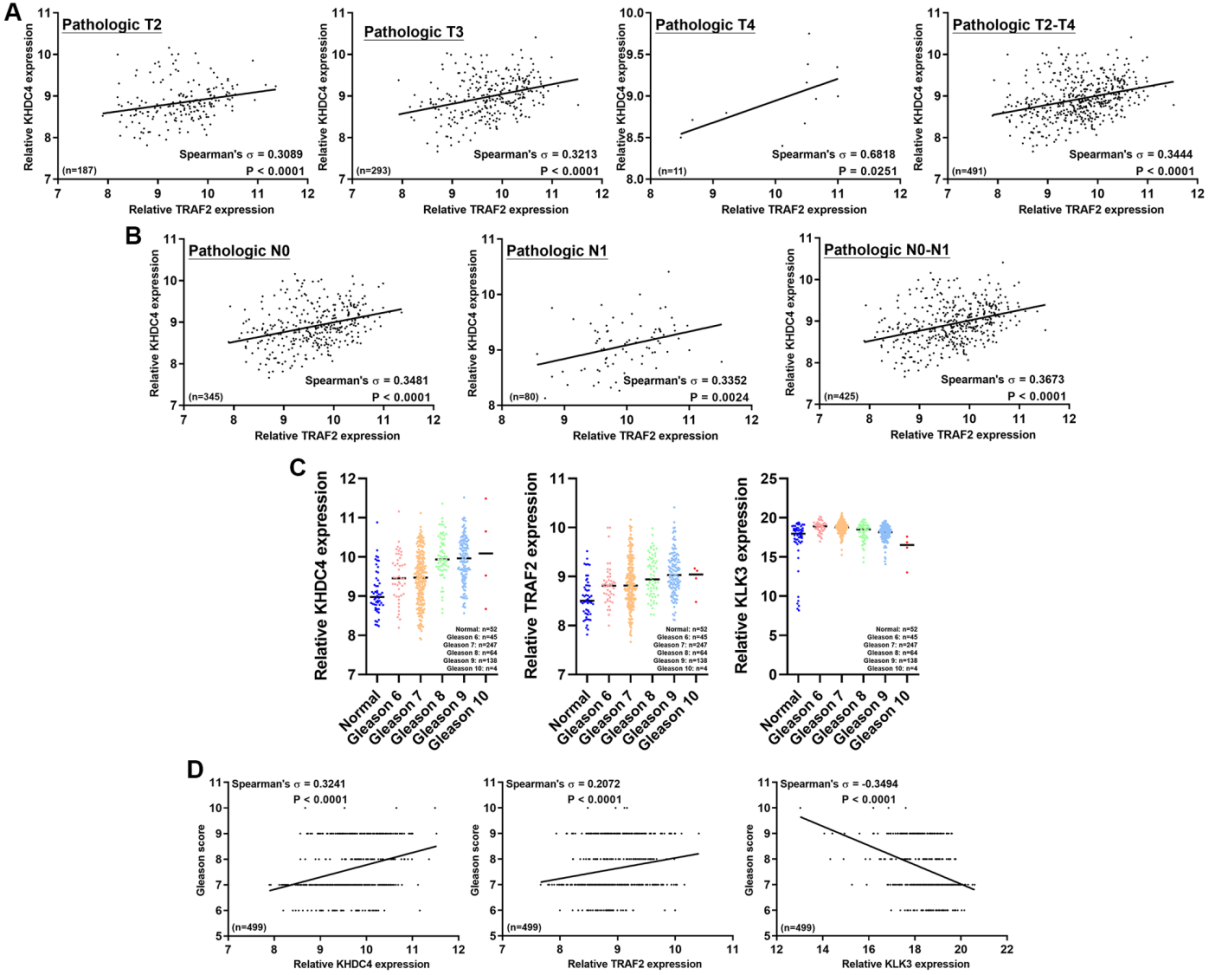
**The correlation between KHDC4 and TRAF2 at a pathological level contributes to an advanced Gleason score.** (**A**) The correlation between KHDC4 and TRAF2 in distinct pathologic stages of TCGA-PRAD data. (**B**) The correlation between KHDC4 and TRAF2 in different pathologic lymph node metastasis stages of TCGA-PRAD data. (**C**) The related levels of KHDC4, TRAF2, and KLK3 in TCGA-PRAD data across various Gleason scores. (**D**) The correlation of KHDC4, TRAF2, and KLK3 with Gleason scores in TCGA-PRAD data.

To further confirm whether the results between KHDC4 and TRAF2 in PCa patients can serve as a prognosis factor in different pathologic stages, a series of correlations were conducted. In the early pathologic T2 stage, KHDC4 showed a positive correlation with TRAF2 (Spearman’s correlation = 0.3089, *p* < 0.0001). Similar positive correlations were observed in pathologic T3 stage (Spearman’s correlation = 0.3213, *p* < 0.0001), pathologic T4 stage (Spearman’s correlation = 0.6818, *p* = 0.0251), and pathologic T2-T4 stage (Spearman’s correlation = 0.3444, *p* < 0.0001) (Figure 3A). Regarding lymph node metastasis, in pathologic N, KHDC4 exhibited a positive correlation with TRAF2 (Spearman’s correlation = 0.3481, *p* < 0.0001). Positive correlations were also observed in pathologic N1 stage (Spearman’s correlation = 0.3352, *p* < 0.0024) and pathologic N0-N1 stage (Spearman’s correlation = 0.3673, *p* < 0.0001) (Figure 3B) (Supplementary Table 4). Furthermore, the Gleason grading system was used to assess the correlation between KHDC4 and TRAF2 in PCa biopsy stages. Importantly, the levels of KHDC4 and TRAF2 increased with rising Gleason scores, in contrast to using KLK3 (PSA) as a prognosis factor, which did not exhibit a positive trend (Figure 3C) (Supplementary Table 10). In a more detailed analysis, the correlation results indicated that KHDC4 (Spearman’s correlation = 0.3241, *p* < 0.0001) and TRAF2 (Spearman’s correlation = 0.2072, *p* < 0.0001) were positively correlated with Gleason score, while a negative correlation was observed with KLK3 (Spearman’s correlation = −0.3494, *p* < 0.0001) (Figure 3D) (Supplementary Table 10). These findings suggest that utilizing KHDC4 and TRAF2 as prognosis factors in PCa may provide greater practical value than using PSA as a biomarker.

### Common gene ontology regulated by TRAF2 and KHDC4 contributes to a deteriorated prognosis

**Figure 4:**
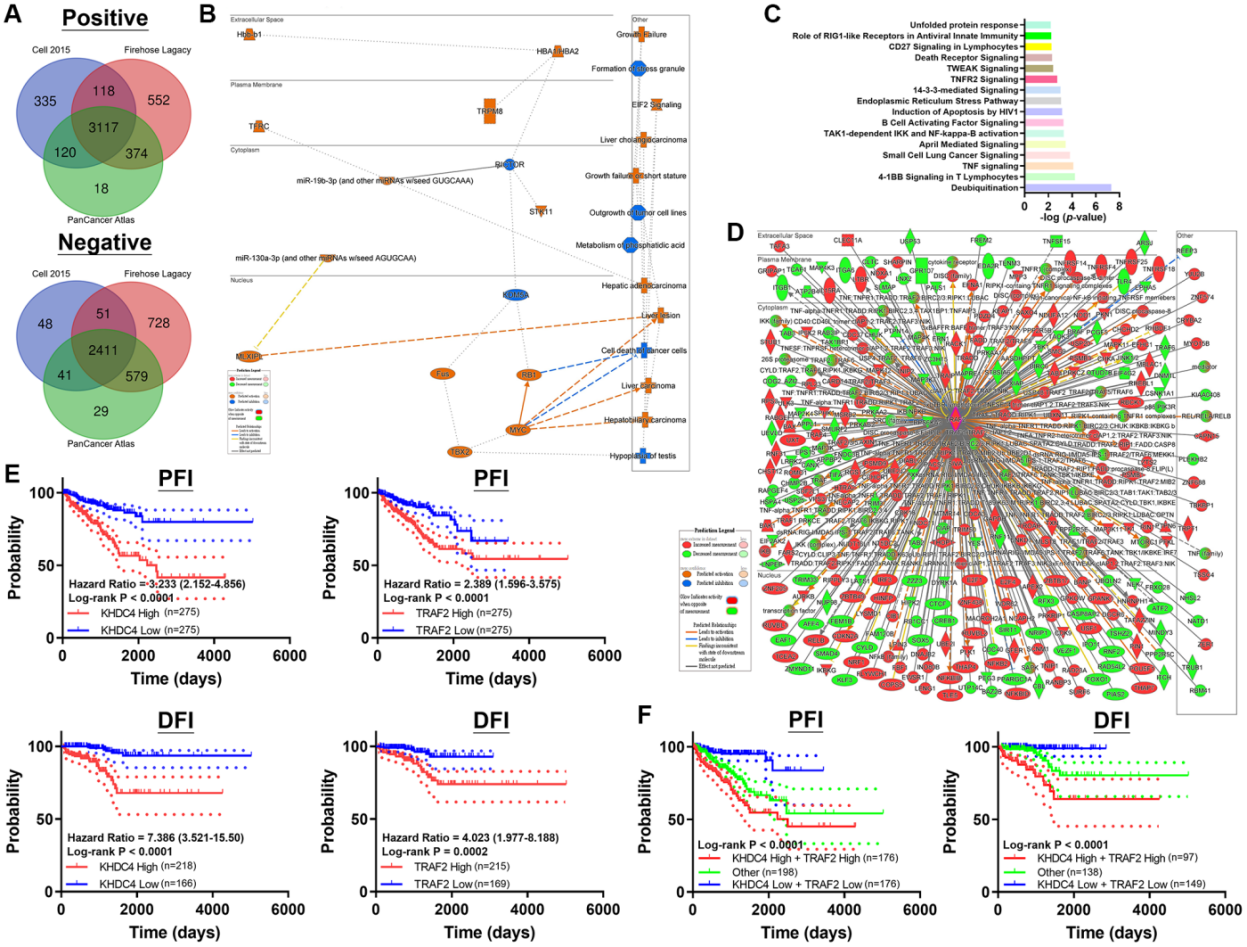
**Shared gene ontology between TRAF2 and KHDC4 is associated with adverse prognosis outcomes.** (**A**) A Venn diagram analysis collecting molecules positively or negatively correlated to TRAF2 in TCGA-PRAD datasets (Cell 2015, Firehose Legacy, and PanCancer Atlas). (**B**) Graphical abstract illustrating the potential biological roles influenced by TRAF2 in prostate cancer. (**C**) Canonical pathways affected by TRAF2-related molecules. (**D**) Molecular links to TRAF2 regulations in prostate cancer. (**E**) The correlation of KHDC4 and TRAF2 individually with Progression-Free Interval (PFI) and Disease-Free Interval (DFI) in TCGA-PRAD. (**F**) The correlation of Progression-Free Interval (PFI) and Disease-Free Interval (DFI) in TCGA-PRAD with the combined distribution of KHDC4 and TRAF2.

To establish a molecular simulated model, molecules positively (Spearman values > +0.3) and negatively (Spearman values > −0.3) correlated with TRAF2 were identified from three TCGA-PRAD datasets (Cell 2015, Firehose Legacy, and PanCancer Atlas). Approximately 3117 positive and 2411 negative molecules correlated with TRAF2 were selected using Venn diagrams (Figure 4A) (Supplementary Table 7). Graphical profiles from Ingenuity Pathway Analysis (IPA) software revealed that TRAF2 primarily influences cellular functions, predominantly participating in functions such as growth and tumorigenesis (Figure 4B). Interestingly, these biological functions, conducted by different signaling transductions by TRAF2, were similar to those of KHDC4 (Figure 4C) (Supplementary Table 8). The molecular interaction of TRAF2 also supported that these correlated molecules have been found to participate in TRAF2 regulation (Figure 4D). Furthermore, the correlation of KHDC4 and TRAF2 with Progression-Free Interval (PFI) and Disease-Free Interval (DFI) values was investigated to provide insight into the correlation between clinical deterioration and recurrence after treatment. The results showed that patients with higher KHDC4 or TRAF2 expression levels in TCGA-PRAD data had poor PFI (KHDC4: *p* < 0.0001, HR = 3.233 (2.152–4.8560; TRAF2: *p* < 0.0001, HR = 2.389 (1.596–3.575)) and DFI prognosis values (KHDC4: *p* < 0.0001, HR = 7.386 (3.521–15.50); TRAF2: *p* < 0.0001, HR = 4.023 (1.977–8.188)) (Figure 4E). Importantly, combining the prognosis values of KHDC4 and TRAF2 revealed that patients with high KHDC4 and high TRAF2 levels were significantly distinguished with the worst PFI and DFI values compared to patients with low KHDC4 and low TRAF2 (Figure 4F). These results indicate that the correlation between KHDC4 and TRAF2 in PCa involves regulating similar gene ontologies, contributing to worse prognosis associated with malignancy and recurrence.

### The regulatory control of the KHDC4-TRAF2 axis involves E2F4 as an upstream modulator

**Figure 5:**
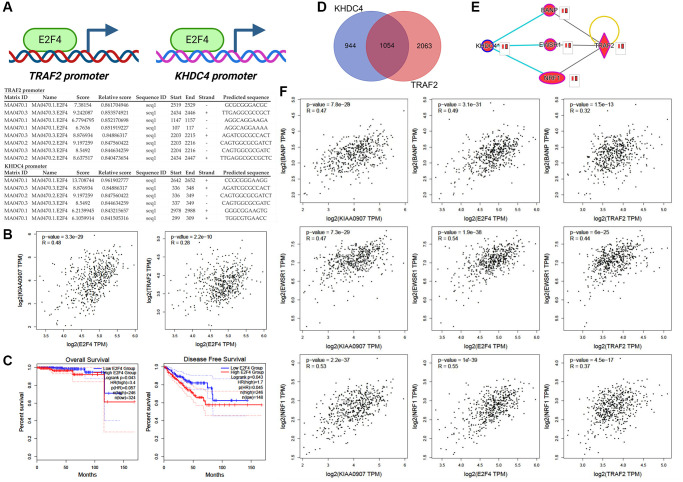
**E2F4 is positioned as an upstream controller in the regulatory cascade of KHDC4 and TRAF2.** (**A**) Promoter assays outlining potential E2F4 binding sequences in the promoter sequences of TRAF2 and KHDC4. (**B**) The correlation of E2F4 with KHDC4 or TRAF2 in TCGA-PRAD. (**C**) The prognosis values of E2F4 on overall survival or disease-free survival rate in prostate cancer. (**D**) A Venn diagram analysis collecting molecules positively correlated to KHDC4 and TRAF2 in TCGA-PRAD datasets (Cell 2015, Firehose Legacy, and PanCancer Atlas). (**E**) Downstream effectors co-regulated by KHDC4 and TRAF2. (**F**) The correlation of downstream effectors of KHDC4 and TRAF2 with E2F4 in TCGA-PRAD.

To investigate the potential regulation between KHDC4 and TRAF2, an upstream regulators analysis was performed using IPA software, revealing E2F4 as a potential activated transcription factor for TRAF2 transactivation (Supplementary Figure 5). Analysis of the TRAF2 promoter, approximately 3000bp upstream, through JASPAR datasets, identified eight potential E2F4 binding sites with an 84% relative profile score threshold (Figure 5A). Surprisingly, a similar analysis of the KHDC4 promoter also revealed six potential E2F4 binding sites. In TCGA-PRAD, E2F4 showed a positive correlation with KHDC4 (Spearman's correlation = 0.48, *p* = 3.3E-29) and TRAF2 (Spearman’s correlation = 0.28, *p* = 2.2E-10) (Figure 5B). In PCa, higher expression levels of E2F4 were associated with worse overall survival prognosis values (*p* = 0.043, HR = 3.4) and disease-free survival (*p* = 0.043, HR = 1.7) (Figure 5C). The involvement of E2F4 in cell cycle regulation within PCa has been documented in earlier studies [26]. To investigate this in our models, we generated E2F4 knockdown in C4-2 and PC-3 cell lines (Supplementary Figure 6A, 6B). Colony formation and proliferation assays confirmed that E2F4 plays a consistent role in promoting tumor growth in PCa (Supplementary Figure 6C, 6D). Additionally, knockdown of E2F4 significantly reduced wound healing capacity (Supplementary Figure 6E) and impaired the invasive potential of the cells, as shown by transwell migration assays (Supplementary Figure 6F, 6G). To explore whether E2F4 regulates the transcription of KHDC4 and TRAF2, we performed qPCR assays, revealing that E2F4 predominantly influences TRAF2 transcription in androgen receptor-positive C4-2 cells, while notably decreasing KHDC4 levels in androgen receptor-negative PC-3 cells (Supplementary Figure 7A). Treatment with the E2F4 inhibitor HLM006474 produced comparable effects (Supplementary Figure 7B). As an upstream regulator, E2F4 was found to influence the transactivation of KHDC4 and TRAF2, subsequently leading to decreased protein levels in PCa cells, as evidenced by immunoblotting (Supplementary Figure 7C). To further determine whether E2F4 regulates KHDC4 and TRAF2 in an androgen receptor-dependent manner, we employed both androgen receptor-positive 22RV1 cells and androgen receptor-negative DU145 cells. qPCR analysis demonstrated that inhibition of E2F4 via HLM006474 suppressed KHDC4 and TRAF2 expression in 22RV1 cells, mirroring the expression profile observed in PC-3 cells (Supplementary Figure 7B). However, in DU145 cells, E2F4 downregulation resulted in the upregulation of KHDC4 and TRAF2 (Supplementary Figure 8A). Moreover, analysis of human prostate cancer datasets from TCGA (Cell, Firehose Legacy, and PanCancer) did not reveal a significant positive correlation between KHDC4 or TRAF2 and androgen receptor expression (Supplementary Figure 8B). These findings suggest that E2F4-mediated transactivation of KHDC4 and TRAF2 occurs independently of androgen receptor signaling. Furthermore, combining E2F4 with either KHDC4 or TRAF2 resulted in even worse overall survival rate (E2F4+KHDC4: *p* ≤ 0.025, HR = 5; E2F4+TRAF2: *p* < 0.029, HR = 4.8) (Supplementary Figure 9A) and disease-free survival rate (E2F4+KHDC4: *p* ≤ 0.0001, HR = 2; E2F4+TRAF2: *p* < 7.3E-05, HR = 2.3) (Supplementary Figure 9B). These results suggest that E2F4 transactivation of KHDC4 or TRAF2 may contribute to increased malignancy in PCa. By combining KHDC4 and TRAF2 positive-correlated molecules from three TCGA-PRAD datasets, approximately 1054 positive molecules (Spearman values > +0.3) were identified using a Venn diagram (Figure 5D) (Supplementary Table 11). The molecular interaction network indicated that BANP, EWSR1, and NRF1 were co-regulated by KHDC4, TRAF2, and E2F4 according to current publications (Figure 5E). These three molecules showed positive correlations with KHDC4, TRAF2, and E2F4 in PCa patients (Spearman’s correlation > 0.3, p < 0.001) (Figure 5F). Interestingly, beyond the known associated molecules, these 1054 molecules, when considered as signatures (the number of genes, excluding lncRNA, snoRNA, and pseudogenes, is 1026), exhibited positive correlations with KHDC4 (Spearman’s correlation = 0.77, *p* = 4E-96) and TRAF2 (Spearman’s correlation = 0.61, *p* = 1.6E-51) (Supplementary Figure 10A). Combining KHDC4 and TRAF2 with these downstream effectors as a signature also showed a positive correlation to E2F4 in TCGA-PRAD datasets (Spearman’s correlation = 0.43, *p* = 3.6E-23). Moreover, this signature can be used for prognosis on overall survival (*p* = 0.048, HR = 4.3) and disease-free survival rate (*p* = 3.8E-07, HR = 3.1) (Supplementary Figure 10B). These results indicate that E2F4, as a potential upstream regulator of the KHDC4-TRAF2 axis, leads to worse prognosis values. The KHDC4-TRAF2 axis, in addition to serving as a new biomarker, may use their mediated downstream effectors as a signature for a useful prognosis in advanced PCa.

## DISCUSSION

The utilization of prostate-specific antigen (PSA) as a screening biomarker has significantly improved the mortality rate of PCa since 1986 [27]. However, despite using a PSA concentration below 4 ng/mL as the normal threshold, approximately 15% of the population is still diagnosed with advanced PCa [28]. Moreover, the rise in PSA levels is influenced by factors such as benign prostatic enlargement, age, and prostatitis, making it less tumor-specific [29]. Therefore, there is a necessity to identify novel biomarkers for PCa prognosis. In this study, profiling analysis of transcriptome datasets between late-stage and early-stage PCa identified KHDC4 as a member of the KH Homology Domain-Containing Protein family with higher expression in prostate tissues, further elevated in tumor groups (Figure 1B). Systematic analysis results from both TCGA and GEO databases demonstrated that increased KHDC4 in PCa tissues is associated with malignancy, including advanced stages and metastasis activity. Compared to other upregulated molecules identified in the sequencing profiles of patients with advanced-stage PCa (Supplementary Table 1), higher KHDC4 transcript levels were significantly associated with poorer overall survival and disease-free survival outcomes, underscoring KHDC4 as a valuable prognostic factor in PCa (Figure 1). However, without further histopathological validation, the prognostic relevance of protein levels for these other upregulated molecules in PCa patients cannot be excluded either. Through a KHDC4-based molecular simulated model [21–24], abnormal elevation of KHDC4 levels was implicated in participating in epithelial proliferation and repair-related functions in tumor cells. This involvement may occur through the regulation of signaling pathways such as endoplasmic reticulum stress and unfolded protein response (Figure 2C). In colorectal cancer, an increase in KHDC4 was associated with GALC expression in patients treated with oxaliplatin and capecitabine [8]. In this study, the knockdown of KHDC4 supports its role in contributing to tumor growth and motility features in the PCa cell model (Figure S2). Given that TRAF2 is known to respond to stress and attenuate apoptosis events, the KHDC4-mediated gene ontology also consistently observed TRAF2 involvement in multiple signaling pathways (Figure 2C) (Supplementary Table 8). Thus, KHDC4 may serve as an environmental stress response factor. Although the molecules involved in the gene ontology mediated by KHDC4 exhibited significant correlations according to Spearman analysis, their prognostic significance was not as pronounced as that observed for TRAF2. Moreover, single-cell profiling indicated that although the expression intensity of KHDC4 and TRAF2 in malignant cells was higher than in other clusters or cell types, the differences in the proportion of positive cells may be attributed to distinct tumor cell populations. This suggests that the regulatory relationship between KHDC4 and TRAF2 might not be direct at the cellular level, with paracrine, endocrine, or autocrine signaling potentially playing a role (Figure 2K). Consistent with previous findings [25], the knockdown of TRAF2 in PCa cells demonstrated effects on biological functions that align with its established role in promoting tumor growth and malignant activity (Supplementary Figure 4). The molecular simulated model, however, could not confirm whether KHDC4 can activate TRAF2 through E2F4 in response to stress. Nevertheless, the KHDC4-based molecular interaction model showed that other molecules are reported to be associated with drug stimulation (Figure 2D). Induction of ZRSR2 was found to contribute to the development of castration-resistant PCa during Androgen deprivation therapy [30]. The expression of NRF1 has been reported to be associated with a response to oxidative stress and androgen signaling [31, 32]. Therefore, these molecules associated with KHDC4 suggest that KHDC4 can serve as a molecular response to environmental stimuli in PCa. Analyzing these signaling pathways in more detail, TRAF2 was found to be involved in all the KHDC4-mediated gene ontologies, although with different ranking orders. However, in the TRAF2-based simulated molecular interaction model, these signaling scores significantly increased, indicating a more extensive involvement of molecules in TRAF2 regulation (Figure 4C). Thus, it is speculated that KHDC4 primarily influences these signaling pathways and biological functions, especially environmental stress, through TRAF2. Considering past findings that TRAF2 is associated with poor prognosis in PCa (Figure 2L) [33], the increased levels of KHDC4 and TRAF2, observed in both TCGA and GEO databases, positively correlated in advanced PCa patients. This correlation was also observed in CCLE-PCa cell lines and tumor microenvironments (Figure 2G and 2K). Importantly, the correlation between KHDC4 and TRAF2 among different pathologic tumor stages and lymph node metastasis was reflected in the Gleason score evaluation system based on PCa tissue biopsy. Compared to using PSA (LKL3), the levels of KHDC4 and TRAF2 significantly increased with rising Gleason scores and exhibited positive correlations (Figure 3), supporting the potential of the KHDC4-TRAF2 axis as a biomarker for prognosis purposes in PCa. Interestingly, the molecular simulated model analysis revealed that TRAF2 and KHDC4 can influence similar gene ontologies to regulate PCa malignancy (Figure 2C and 4C) (Supplementary Table 8). The Venn diagram and IPA analysis showed that KHDC4 and TRAF2 can regulate similar downstream effectors, including BANP, EWSR-1, and NRF1 (Figure 5E). In TCGA-PRAD, BANP, EWSR-1, and NRF1 were positively correlated with KHDC4 and TRAF2 (Figure 5F). Although the molecular interaction relationship can be obtained through software analysis, the roles of BANP and EWSR-1 in PCa remain unknown. Overexpression of NRF1 is known to be associated with the growth and motility of PCa [34], supporting the link between the E2F4-transactivated KHDC4 and TRAF2 axis and metastasis activity. Additionally, this study identified 1051 molecules not previously associated with KHDC4 or TRAF2 (Figure 5D) (Supplementary Table 11). These downstream effectors exhibited significant positive correlations with KHDC4 or TRAF2 and served as a prognosis signature for overall survival and disease-free survival in PCa (Supplementary Figures 10 and 11). With this molecular correlation support, patients with high expression levels of KHDC4 or TRAF2 were observed to have a significantly correlated progression-free interval (PFI) and disease-free interval (DFI) in clinical outcomes. Patients with both high KHDC4 and high TRAF2 levels showed worse prognostic outcomes (Figure 4E, 4F).

**Figure 6:**
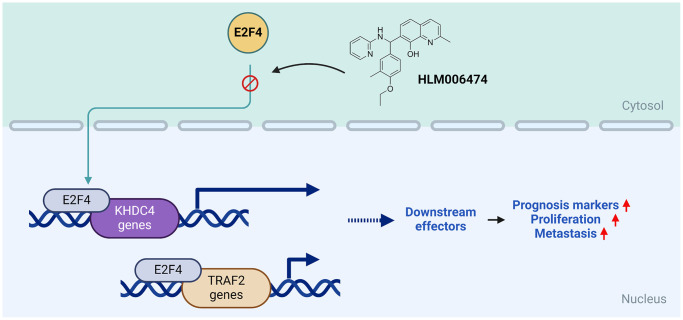
**The diagram illustrates how E2F4 potentially functions as a transcription factor to activate the KHDC4-TRAF2 axis, leading to the generation of downstream effectors and promoting the development of advanced prostate cancer.** The molecules identified in this model can be employed as valuable prognosis biomarkers or signatures for predicting potential outcomes in prostate cancer patients.

According to the molecular simulated model results, E2F4 was identified as an upstream regulator for TRAF2 transactivation, and it was found that KHDC4 also has multiple E2F4 transcription factor binding regions (Figure 5A). The positive correlation between E2F4 and KHDC4 or TRAF2 was observed in TCGA-PRAD profiles (Figure 5B), indicating that the clinical relevance of E2F4 regulating KHDC4 or TRAF2. In the E2F4 knockdown model, E2F4 was confirmed to contribute to tumor malignancy features, including tumor growth and motility (Supplementary Figure 6). Additionally, selective regulation of TRAF2 and KHDC4 was observed in cells with differential androgen receptor expression, as evidenced by qPCR (Supplementary Figure 7A) and immunoblotting (Supplementary Figure 7C) results. Interestingly, treatment with an E2F4 inhibitor also demonstrated a similar regulatory trend (Supplementary Figure 7B). Electrophoretic mobility shift assays have demonstrated that HLM006474 inhibits the DNA-binding ability of E2F4 and downregulates its protein levels in A375 cells [35]. Similarly, treatment with HLM006474 in PCa cells resulted in the suppression of both E2F4 RNA and protein levels, which subsequently altered the transcription of its downstream effectors (Supplementary Figure 7). Notably, the androgen receptor-independent transactivation of KHDC4 and TRAF2 by E2F4 was demonstrated using multiple prostate cancer cell lines (Supplementary Figures 7B and 8A) and further validated through clinically relevant transcriptome datasets (Supplementary Figure 8B). Interestingly, E2F4 also appears to exert a transrepressive effect on KHDC4 and TRAF2 regulation in DU145 cells. Moreover, the downstream effectors of KHDC4 and TRAF2 were observed to be positively correlated with E2F4 (Figure 5B, Supplementary Figure 9A), suggesting that E2F4, in addition to affecting KHDC4 and TRAF2 directly, indirectly influences their downstream regulators to participate in cancer malignancy. These results partially explain why combining E2F4 with KHDC4 or TRAF2 results in even worse overall survival rate (Supplementary Figure 8A) or disease-free survival rate (Supplementary Figure 8B). Subsequently, transactivating KHDC4 or TRAF2 by E2F4 may lead to increased malignancy in PCa. In this study, E2F4 was proposed for the first time as a potential upstream regulator for KHDC4 and TRAF2 in PCa. Overall, the positive correlation between KHDC4 and TRAF2 in PCa can be considered a potential prognostic biomarker. Both the upstream and downstream regulators of KHDC4 and TRAF2 consistently indicate that, apart from participating in tumor malignancy, the molecules centered around the KHDC4-TRAF2 axis can be further utilized as a PCa prognosis signature, as illustrated in Figure 6.

## MATERIALS AND METHODS

### Transcriptomic profiles associated with clinical prostate adenocarcinoma

The expression transcriptome profiles of KHDC4, TRAF2, E2F4, and their associated downstream effectors in prostate cancer were gathered from The Cancer Genome Atlas Program (TCGA) database (Cell 2015, Firehose Legacy, and PanCancer Atlas) (https://www.cancer.gov/ccg/research/genome-sequencing/tcga). Additional transcriptome profiles from cohorts (GSE21032, GSE35988, GSE6919, and GSE176031) of prostate cancer patients were downloaded from the Gene Expression Omnibus (GEO) database (https://www.ncbi.nlm.nih.gov/geo/).

### Molecular simulation model depicting the interaction between KHDC4 and TRAF2

The Venn diagram outcomes were employed to compile molecules based on their positive or negative scores, with a Spearman score exceeding ±0.3, correlated to KHDC4 and TRAF2 across TCGA datasets (Cell 2015, Firehose Legacy, and PanCancer Atlas). Subsequently, these molecules underwent analysis in the Ingenuity Pathway Analysis (IPA) database (https://digitalinsights.qiagen.com/products-overview/discovery-insights-portfolio/analysis-and-visualization/qiagen-ipa/) to generate a graphical abstract, a list of gene ontologies, and the identification of upstream regulators associated with KHDC4 and TRAF2.

### Prognostic assessment and evaluation of molecular correlations

The correlation analysis of KHDC4, TRAF2, E2F4, and their associated downstream effectors in prostate cancer was conducted using the GEPIA2 database (http://gepia2.cancer-pku.cn/#index). This analysis generated correlation scores and prognosis values.

### Identification of upstream regulators

The potential transcription factors of TRAF2 were identified using the IPA database. Approximately 3000bp promoter region sequences of KHDC4 and TRAF2 were downloaded from NCBI (https://www.ncbi.nlm.nih.gov/gene) and subsequently analyzed in the JASPAR database (https://jaspar.elixir.no/) to identify potential transcription factor binding regions.

### Lentivirus and small compound-based gene downregulation model establishment

Human prostate cancer cell lines C4-2, 22RV1, DU145 and PC-3, obtained from ATCC, were cultured under conditions previously described [36]. Briefly, the cells were maintained in RPMI-1640 medium supplemented with 10% fetal bovine serum, 4 mM l-glutamine, and 1% penicillin at 37°C in a 5% CO_2_ atmosphere. Knockdown of KHDC4, TRAF2, and E2F4 was achieved using pLKO.1-shRNA constructs, sourced from the National RNAi Core Facility at Academia Sinica, Taiwan, following the provided lentivirus production protocol. For E2F4 inhibition, PCa cells were treated with 30 μM HLM006474 for 24 hours [35]. The efficiency of both gene knockdown and drug treatment was confirmed via qPCR and immunoblotting, as previously described [22, 37]. The qPCR primers used in this study were as follows: E2F4-F: ACAGTGGTGAGCTCAGTTCA; E2F4-R: GAGGTAGAAGGGTTGGGTCC, TRAF2-F: AGAGCCTGGAGAAGAAGACG; TRAF2-R: CTCCAAGACCTTCTGCTCCA, and KHDC4-F: AGGGCTGGAGTTTGGGATAC; KHDC4-R: CAGGCCCCAGAGTCTTGTTA. Antibodies for KIAA0907 (Catalog No. 25419-1-AP), TRAF2 (Catalog No. 67315-1-Ig), and E2F4 (Catalog No. 67812-1-Ig) were purchased from Proteintech. β-Actin (A5441) was obtained from Sigma-Aldrich. The protein ladder marker used was TD-PM10315 (BIOTOOLS).

### Tumor cells growth and motility biological assessment

The methods for assessing tumor growth and motility in artificially manipulated knockdown cell models were adapted from previous publications [22, 37]. For the colony formation assay, control and experimental tumor cells (~2 × 10^3^ cells/well) were seeded in six-well plates and cultured for two weeks. Cells were then fixed using a methanol-acetic acid solution and stained with crystal violet for visualization. For the cell proliferation assay, cells (~1 × 10^4^ cells/well) were seeded in 96-well plates and monitored over five days. Cell growth was assessed daily using Alamar Blue with absorbance measured at 570 nm. Wound healing assays were conducted using a two-well silicone insert from Ibidi, according to the manufacturer’s protocol. PCa cells (~3 × 10^5^/ml) were seeded in the silicone insert for 24 hours. After removal of the insert, the degree of wound closure was observed in the presence of 10% serum-medium. Tumor cell migration and invasion were evaluated using a Boyden chamber system [22]. Cells were seeded in the upper chamber in serum-free medium after coating with fibronectin (migration) or Matrigel (invasion). The lower chamber contained medium with 10% serum to act as a chemoattractant. Infiltrated cells were fixed with methanol and stained with Giemsa for visualization. Images of experimental data were captured using an optical microscope and analyzed with ImageJ Software for cell quantification.

### Statistical analysis

Differential expression results among different groups in the transcriptome profiles were assessed for statistical significance using unpaired Student’s *t*-tests. Significance levels were indicated by ^*^p < 0.05; ^**^p < 0.01; ^***^p < 0.001. Prognosis analysis generated Cox regression values using IBM SPSS (https://www.ibm.com/products/spss-statistics).

## Supplementary Materials

Supplementary Figures

Supplementary Table 1

Supplementary Table 2

Supplementary Table 3

Supplementary Table 4

Supplementary Table 5

Supplementary Tables 6 and 9

Supplementary Table 7

Supplementary Table 8

Supplementary Table 10

Supplementary Table 11
