# Supplementary material for: Implications of the KHDC4-TRAF2 axis in the context of prostate cancer prognosis
Source: Aging (Albany NY). 2025 Jun 23;17(6):1544–70. doi: 10.18632/aging.206273 (PMC12245195; doi:10.18632/aging.206273)
Supplement: Supplementary Figures [file aging-17-206273-s001.pdf]

SUPPLEMENTARY FIGURES

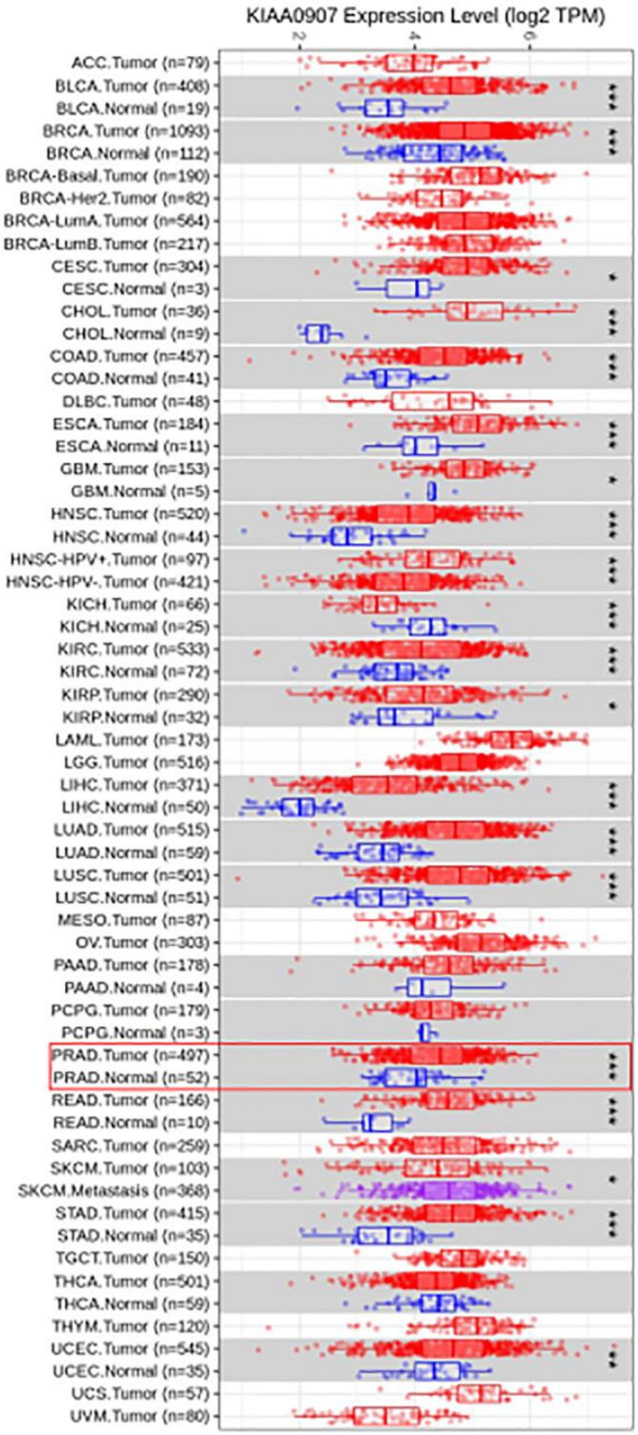

Supplementary Figure 1. The associated expression levels of KHDC4 across various cancer types.

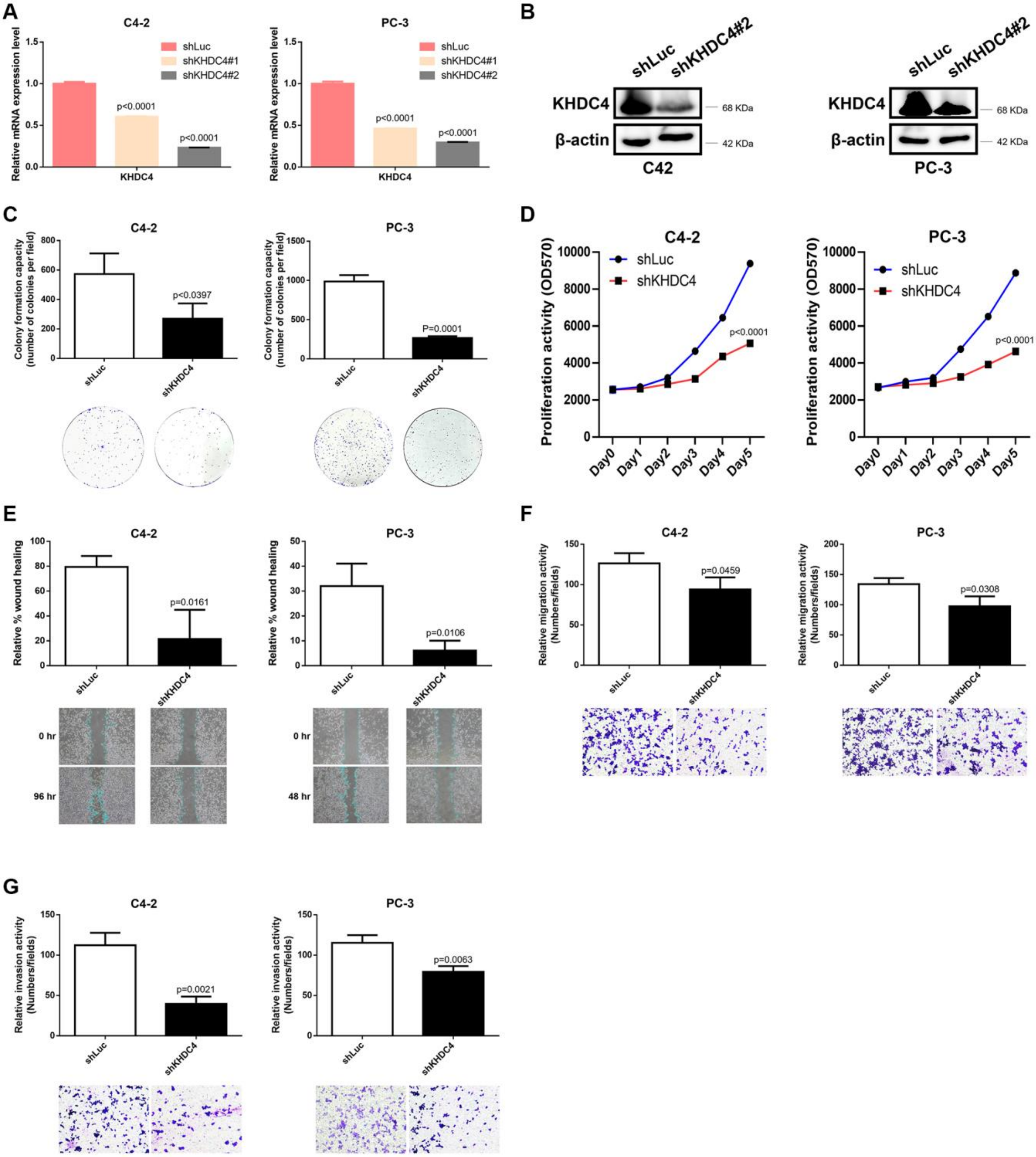

Supplementary Figure 2. The role of KHDC4 depletion in modulating growth and invasion in prostate cancer. (**A**) Quantitative PCR was utilized to validate the effectiveness of lentivirus-mediated KHDC4 knockdown in prostate cancer cell lines. (**B**) KHDC4 expression levels were analyzed through immunoblotting in shLuc and shKHDC4 knockdown cells. (**C**) The effects of KHDC4 knockdown on tumor growth were examined in the C4-2 and PC-3 prostate cancer cell lines. (**D**) The impact of KHDC4 knockdown on cell proliferation was quantified by comparing the proliferation rates of shLuc and shKHDC4-transduced cell lines. (**E**) Wound healing capacity in shLuc and shKHDC4 cells was evaluated to ascertain the impact of KHDC4 expression on wound closure. (**F**) Cell migration ability following KHDC4 knockdown in prostate cancer cells was evaluated through the Boyden chamber assay. (**G**) The Boyden chamber assay was employed to measure changes in invasion capability of prostate cancer cells after KHDC4 knockdown.

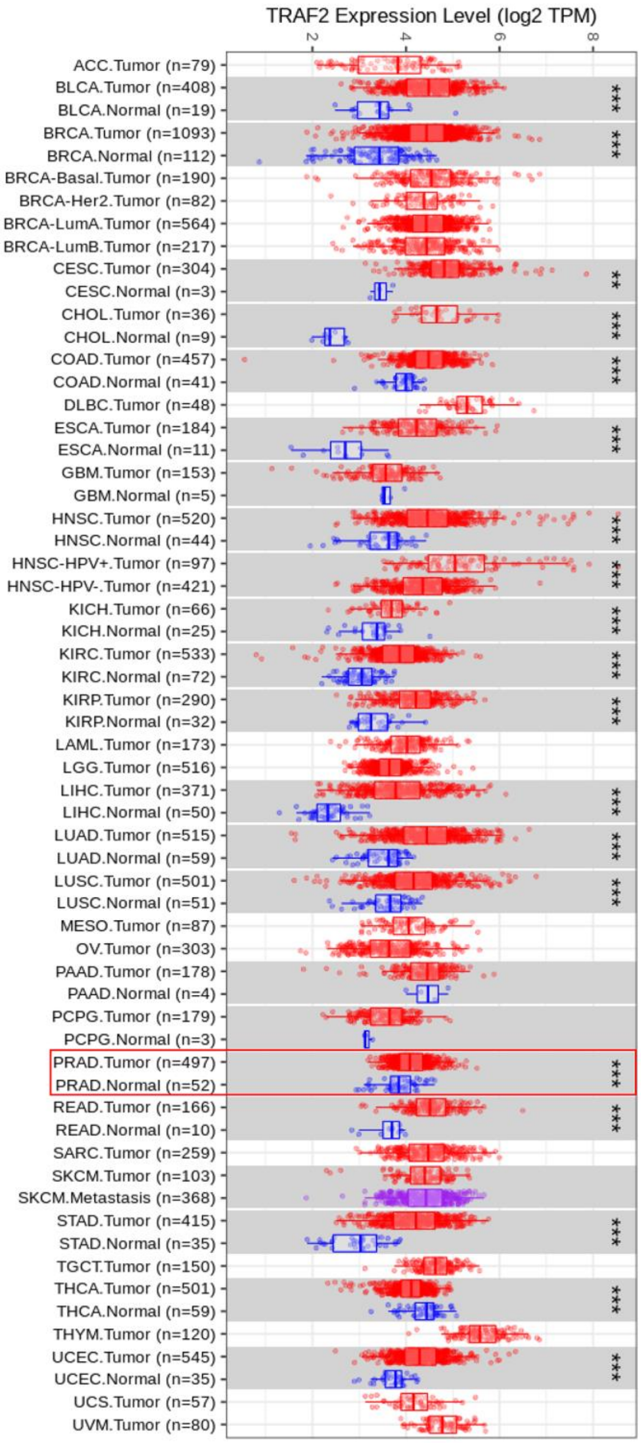

Supplementary Figure 3. The expression patterns of TRAF2 observed in a variety of cancer types.

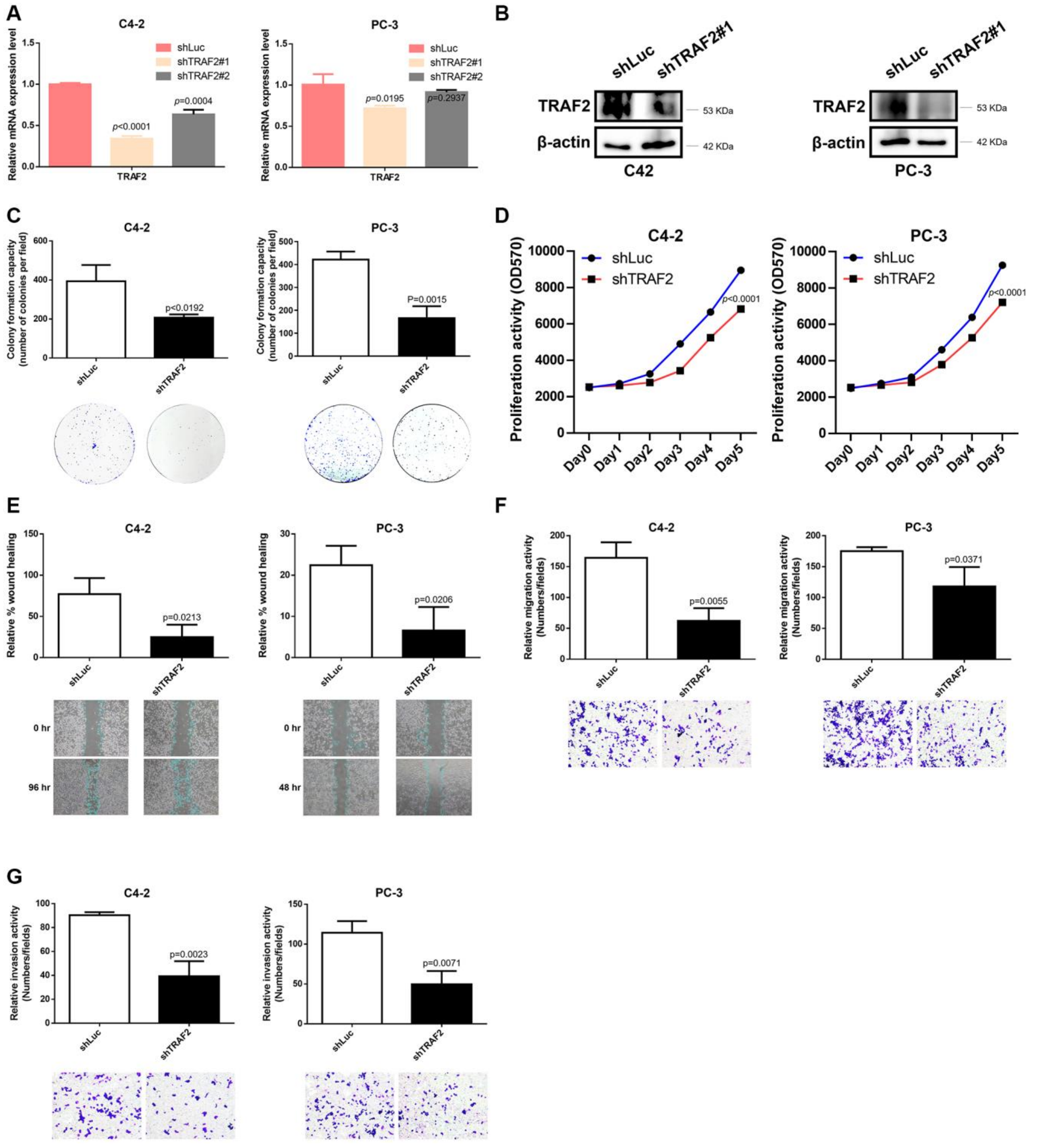

Supplementary Figure 4. Silencing TRAF2 restricts oncogenic traits in human prostate cancer cell lines. (**A**) The efficiency of TRAF2 knockdown by lentiviral transduction in prostate cancer cell lines was confirmed using qPCR. (**B**) The expression of TRAF2 in cells transduced with shLuc or shKHDC4 was determined by immunoblotting. (**C**) TRAF2 knockdown-mediated changes in tumor growth were assessed in C4-2 and PC-3 prostate cancer cells. (**D**) The proliferation of shLuc- and shTRAF2-expressing cells was evaluated through quantitative proliferation assays to determine the effects of TRAF2 knockdown. (**E**) The effect of TRAF2 levels on wound closure was assessed using wound healing assays in both shLuc and sh TRAF2-transduced cells. (**F**) Changes in prostate cancer cell migration due to TRAF2 knockdown were investigated through the Boyden chamber assay. (**G**) TRAF2 knockdown's effect on the invasion potential of prostate cancer cells was quantified using the Boyden chamber assay.

Upstream Regulator	Molecule Type	Predicted Activation State	Activation z-score	*p*-value of overlap	Target Molecules in Dataset
GABA	chemical - endogenous mammalian	Inhibited	-2.292	0.00127	TRAF2
TRAPPC1	other		-1.698	0.0014	TRAF2
E2F4	transcription regulator		1.41	0.00105	TRAF2
POU5F1	transcription regulator	Activated	2.685	0.253	TRAF2
STAT5A	transcription regulator	Inhibited	-2.169	1	TRAF2

Supplementary Figure 5. The simulated model based on KHDC4 identified multiple potential upstream regulators for TRAF2.

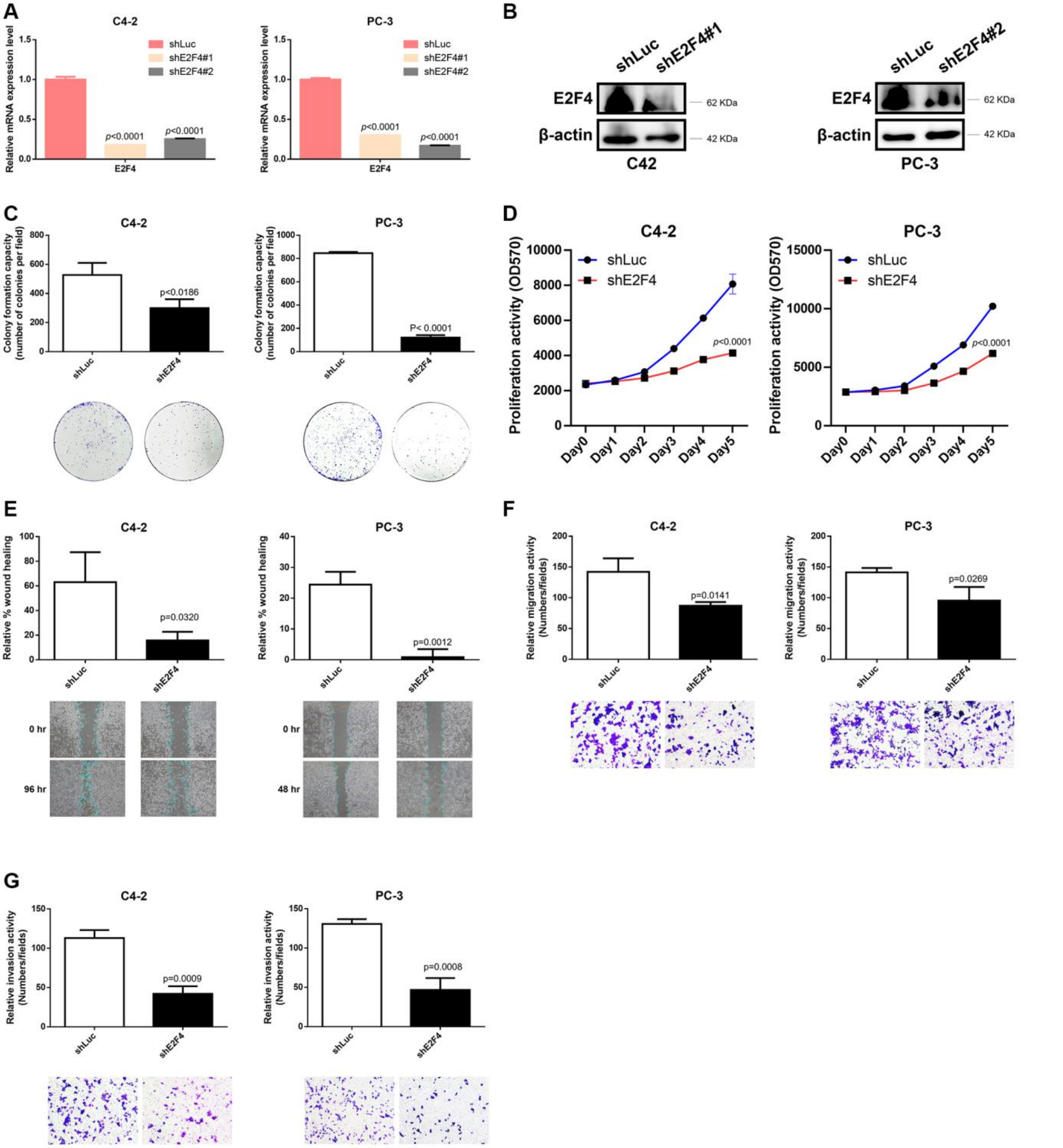

Supplementary Figure 6. E2F4 gene suppression impairs tumor growth and cellular motility in prostate cancer models. (**A**) qPCR was employed to assess the extent of E2F4 suppression following lentiviral transduction in prostate cancer cells. (**B**) Immunoblotting was employed to detect E2F4 protein expression in cells subjected to shLuc or shKHDC4 transduction. (**C**) The impact of reducing E2F4

expression on tumor growth was analyzed in both C4-2 and PC-3 cell lines. (**D**) Proliferation assays were utilized to measure and compare the cell growth rates of shLuc- and shE2F4-transduced cells. (**E**) The role of E2F4 in wound healing was evaluated by conducting wound healing assays in cells with either shLuc or shE2F4 expression. (**F**) The Boyden chamber assay was used to measure changes in migration ability of prostate cancer cells upon E2F4 knockdown. (**G**) Changes in the invasion ability of prostate cancer cells due to E2F4 knockdown were investigated using the Boyden chamber assay.

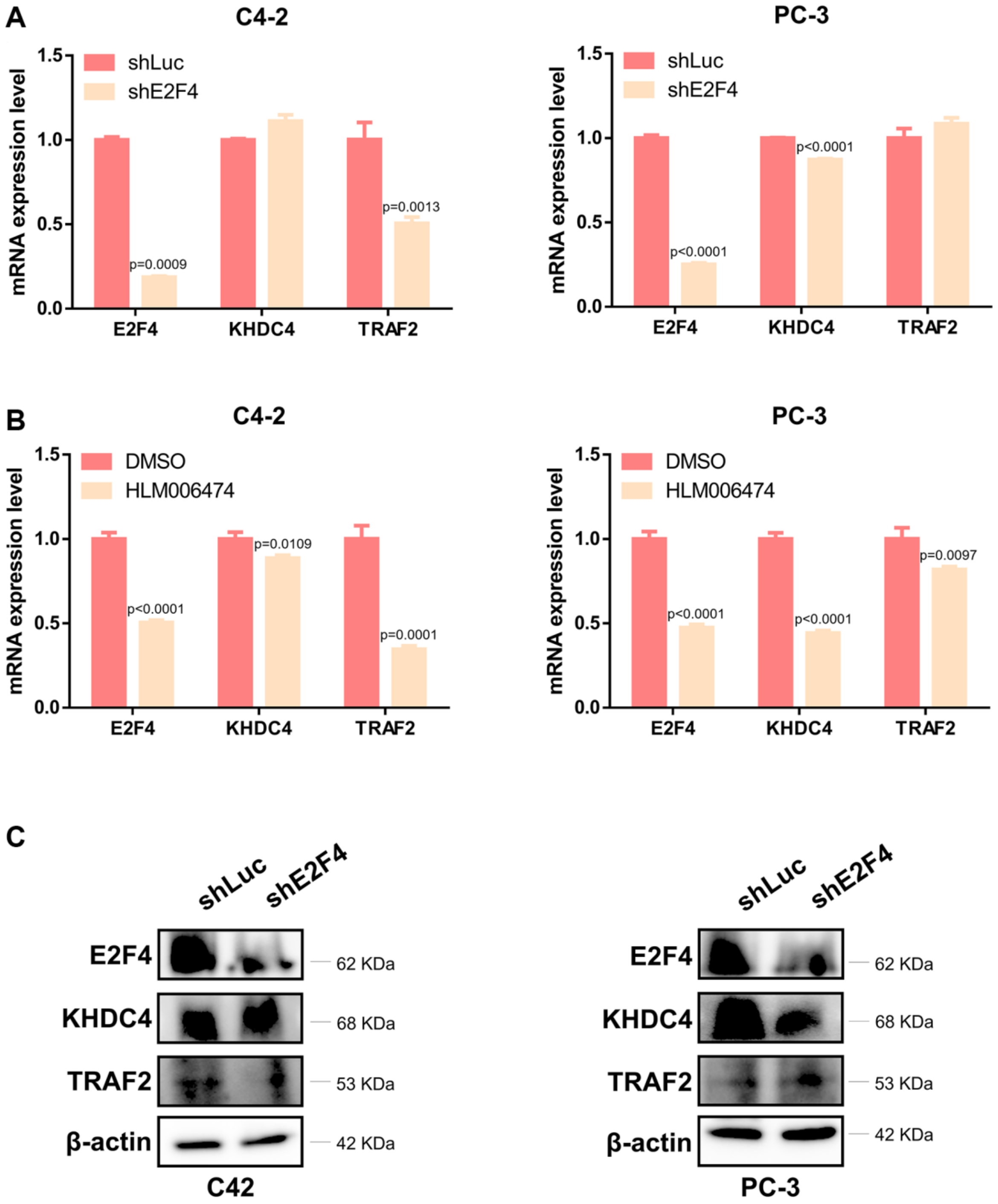

Supplementary Figure 7. The expression levels of KHDC4 and TRAF2 in prostate cancer are modulated by E2F4. (**A**) The influence of E2F4 knockdown on KHDC4 and TRAF2 transactivation in prostate cancer was determined through qPCR analysis. (**B**) The effect of the E2F4 DNA binding inhibitor HLM006474 on KHDC4 and TRAF2 transcription levels in prostate cancer cells was measured by qPCR. (**C**) Immunoblotting was employed to determine the impact of E2F4 on the protein levels of KHDC4 and TRAF2 in prostate cancer cells.

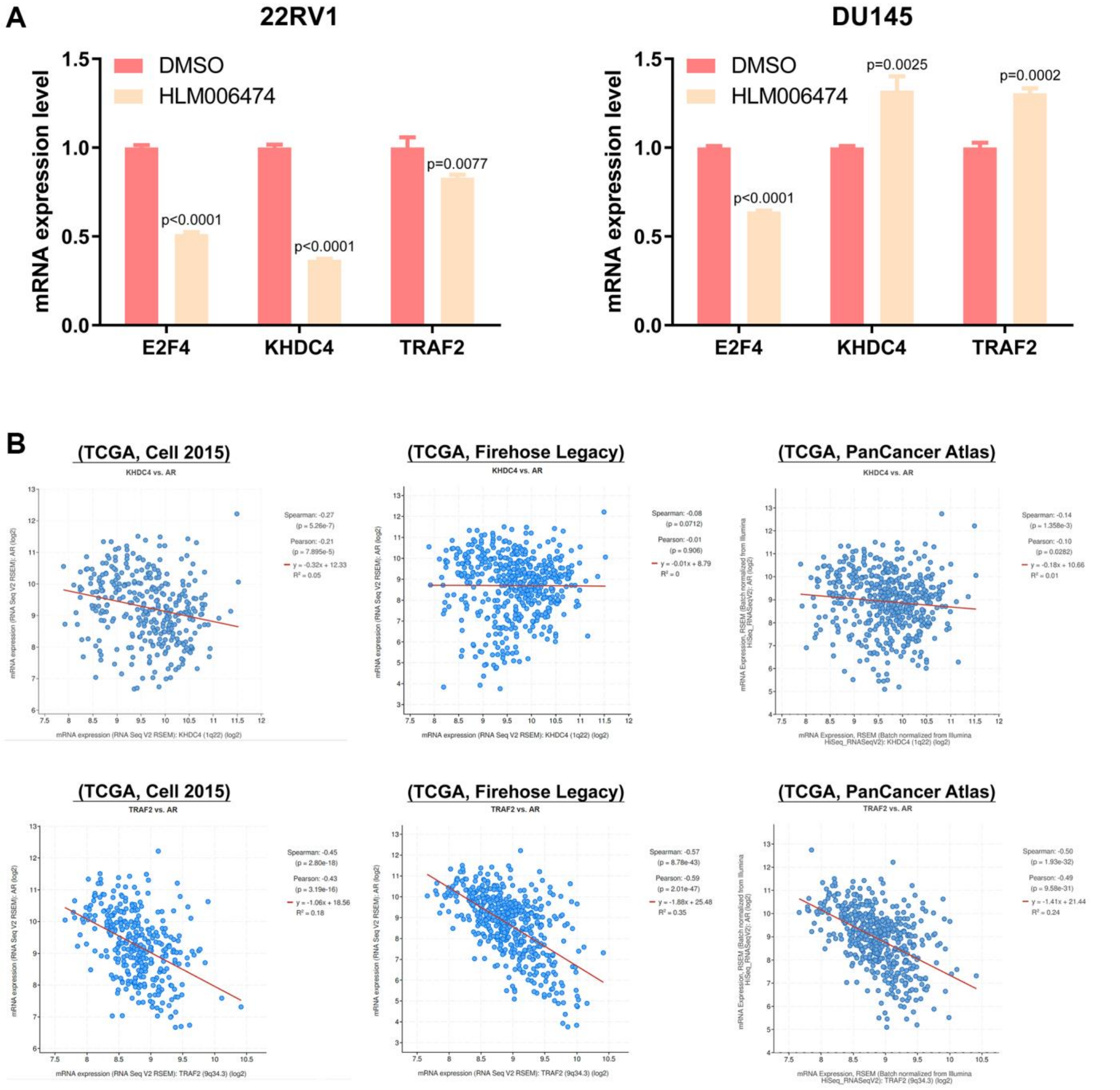

Supplementary Figure 8. E2F4-mediated regulation of KHDC4 and TRAF2 in prostate cancer occurs independently of AR signaling. (**A**) KHDC4 and TRAF2 transcriptional responses to the E2F4 inhibitor HLM006474 in prostate cancer cells were measured by qPCR. (**B**) The co-expression patterns of KHDC4, TRAF2, and the androgen receptor were investigated within the TCGA-PRAD datasets Cell, Firehose Legacy, and PanCancer.

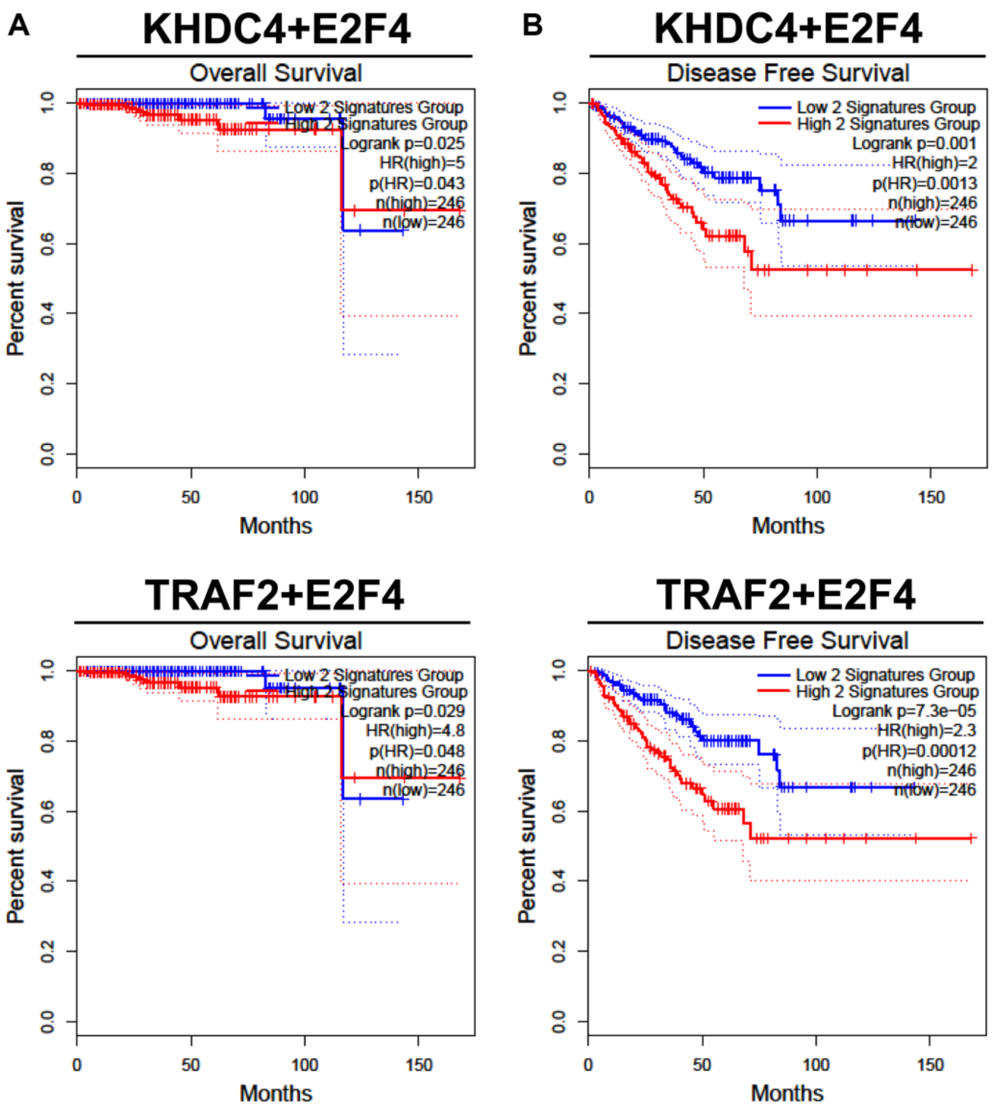

Supplementary Figure 9. The impact of E2F4 as an upstream regulator of KHDC4 and TRAF2 on prognosis in prostate cancer. (**A**) The impact of the combination with E2F4 on the overall survival rate of KHDC4 and TRAF2. (**B**) The influence of the combination with E2F4 on the disease-free survival of KHDC4 and TRAF2.

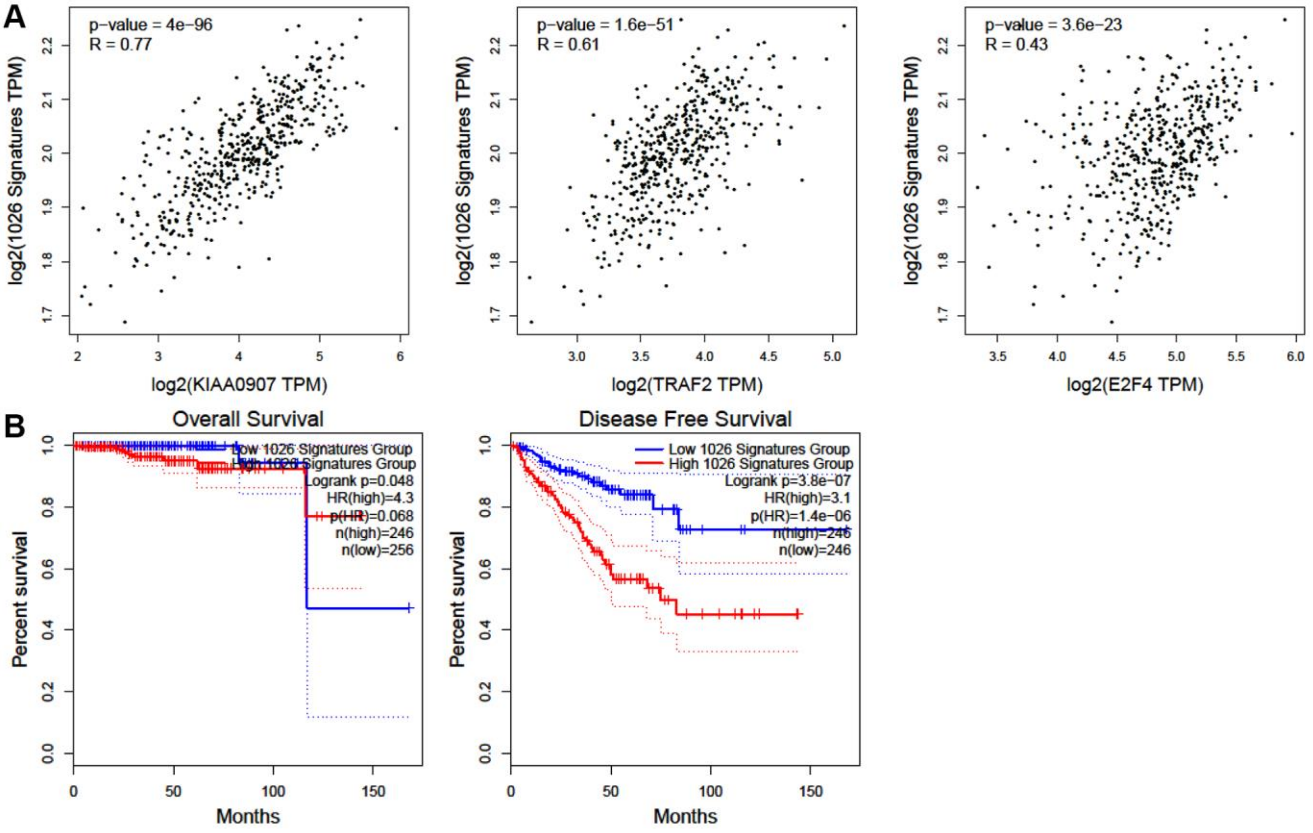

Supplementary Figure 10. The correlation of downstream effectors from the KHDC4-TRAF2 axis serving as signatures for the prognosis of prostate cancer. (**A**) The correlation between KHDC4, TRAF2, and E2F4 with downstream effectors from the KHDC4-TRAF2 axis in TCGA-PRAD. (**B**) The prognosis values of downstream effectors from the KHDC4-TRAF2 axis in TCGA-PRAD.

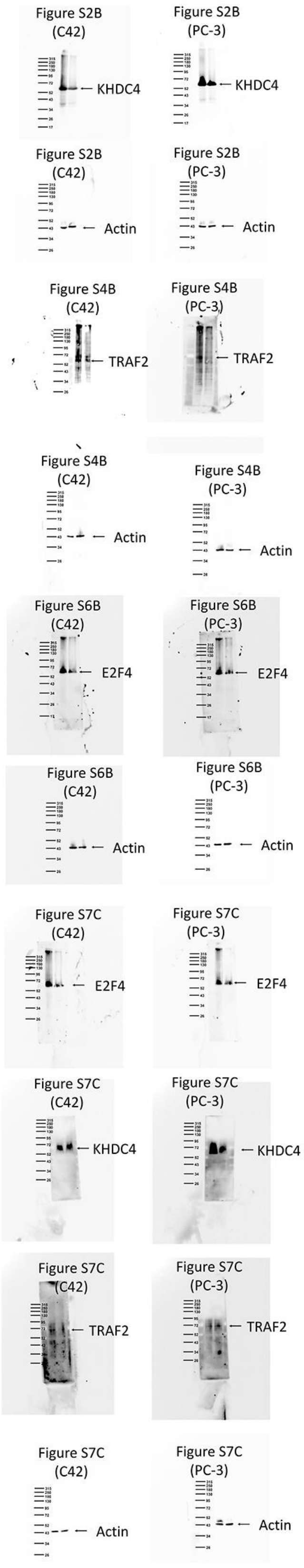

Supplementary Figure 11. The raw data corresponding to the immunoblotting results presented in this study have been provided.
