# Supplementary material for: Implications of the KHDC4-TRAF2 axis in the context of prostate cancer prognosis
Source: Aging (Albany NY). 2025 Jun 23;17(6):1544–70. doi: 10.18632/aging.206273 (PMC12245195; doi:10.18632/aging.206273)
Supplement: Supplementary Tables 6 and 9 [file aging-17-206273-s007.pdf]

SUPPLEMENTARY TABLES

Supplementary Table 6. Correlation of KHDC4 expression in different cancers with prognosis.

Dataset	Cancer type	Endpoint	Probe ID	N	Cox *P*-Value	HR (95% Cilow – Ciupp)
GSE22138	Eye cancer	Distant Metastasis Free Survival	230028_at	63	0.000005	3.24 (1.96–5.38)
GSE4412-GPL97	Brain cancer	Overall Survival	230028_at	74	0.000311	0.22 (0.09–0.50)
GSE2658	Blood cancer	Disease Specific Survival	202220_at	559	0.000708	3.54 (1.70–7.34)
GSE4271-GPL96	Brain cancer	Overall Survival	202220_at	77	0.001858	4.54 (1.75–11.76)
GSE30929	Soft tissue cancer	Distant Recurrence Free Survival	202220_at	140	0.006484	1.77 (1.17–2.66)
GSE17710	Lung cancer	Overall Survival	10376	56	0.006624	0.40 (0.21–0.77)
GSE17537	Colorectal cancer	Disease Free Survival	230028_at	55	0.006874	0.25 (0.09–0.68)
GSE17537	Colorectal cancer	Overall Survival	230028_at	55	0.008948	0.31 (0.13–0.74)
GSE5287	Bladder cancer	Overall Survival	202220_at	30	0.010998	0.32 (0.13–0.77)
GSE17710	Lung cancer	Relapse Free Survival	10376	56	0.012073	0.46 (0.25–0.84)
GSE17537	Colorectal cancer	Disease Specific Survival	230028_at	49	0.0163	0.23 (0.07–0.76)
GSE17710	Lung cancer	Relapse Free Survival	29802	56	0.016317	0.49 (0.27–0.88)
GSE8841	Ovarian cancer	Overall Survival	16646	81	0.017736	5.12 (1.33–19.75)
GSE17536	Colorectal cancer	Overall Survival	202220_at	177	0.018268	2.05 (1.13–3.73)
GSE17710	Lung cancer	Overall Survival	29802	56	0.020272	0.49 (0.27–0.89)
MGH-glioma	Brain cancer	Overall Survival	33885_at	50	0.027691	3.15 (1.13–8.74)
GSE17536	Colorectal cancer	Disease Specific Survival	202220_at	177	0.039201	2.06 (1.04–4.08)
GSE9891	Ovarian cancer	Overall Survival	202220_at	278	0.040115	1.51 (1.02–2.24)
GSE11595	Esophagus cancer	Overall Survival	729937	34	0.045063	4.00 (1.03–15.48)
GSE16560	Prostate cancer	Overall Survival	DAP3_1225	281	0.048955	1.21 (1.00–1.47)

Supplementary Table 9. Related expression of KHDC and TRAF2 in prostate cancer cell lines.

Depmap ID	KHDC4 Gene Effect (DEMETER2) RNAi (Achilles, DEMETER2)	TRAF2 Gene Effect (DEMETER2) RNAi (Achilles, DEMETER2)	Primary disease	Cell line name	Lineage	Primary_disease
ACH-000979	0.256586324	0.100725037	Prostate Adenocarcinoma	DU145	Prostate	Prostate Adenocarcinoma
ACH-000977	0.060982617	−0.000962466	Prostate Adenocarcinoma	LNCAPCLONEFGC	Prostate	Prostate Adenocarcinoma
ACH-000952	0.368814511	0.190593519	Prostate Adenocarcinoma	MDAPCA2B	Prostate	Prostate Adenocarcinoma
ACH-000090	0.217512898	0.118068124	Prostate Adenocarcinoma	PC3	Prostate	Prostate Adenocarcinoma
